# Age-dependent effect of Alzheimer’s risk variant of *CLU* on EEG alpha rhythm in non-demented adults

**DOI:** 10.3389/fnagi.2013.00086

**Published:** 2013-12-13

**Authors:** Natalya Ponomareva, Tatiana Andreeva, Maria Protasova, Lev Shagam, Daria Malina, Andrei Goltsov, Vitaly Fokin, Andrei Mitrofanov, Evgeny Rogaev

**Affiliations:** ^1^Brain Research Department, Research Center of Neurology Russian Academy of Medical ScienceMoscow, Russia; ^2^Vavilov Institute of General Genetics, Russian Academy of SciencesMoscow, Russia; ^3^Center of Brain Neurobiology and Neurogenetics, Institute of Cytogenetics and Genetics, Russian Academy of SciencesNovosibirsk, Russia; ^4^Institute of PsychiatryMoscow, Russia; ^5^University of Massachusetts Medical School, Department of Psychiatry, BNRIWorcester, MA, USA

**Keywords:** Alzheimer’s disease, aging, clusterin, genetic predisposition, EEG, alpha rhythm

## Abstract

Polymorphism in the genomic region harboring the *CLU* gene (rs11136000) has been associated with the risk for Alzheimer’s disease (AD). *CLU C* allele is assumed to confer risk for AD and the *allele T* may have a protective effect. We investigated the influence of the AD-associated *CLU* genotype on a common neurophysiological trait of brain activity (resting-state alpha-rhythm activity) in non-demented adults and elucidated whether this influence is modified over the course of aging. We examined quantitative electroencephalography (EEG) in a cohort of non-demented individuals (age range 20–80) divided into young (age range 20–50) and old (age range 51–80) cohorts and stratified by *CLU* polymorphism. To rule out the effect of the apolipoprotein E *(ApoE)* genotype on EEG characteristics, only subjects without the *ApoE ε4* allele were included in the study. The homozygous presence of the AD risk variant *CLU CC* in non-demented subjects was associated with an increase of alpha3 absolute power. Moreover, the influence of *CLU* genotype on alpha3 was found to be higher in the subjects older than 50 years of age. The study also showed age-dependent alterations of alpha topographic distribution that occur independently of the *CLU* genotype. The increase of upper alpha power has been associated with hippocampal atrophy in patients with mild cognitive impairment (Moretti etal., 2012a). In our study, the *CLU CC-*dependent increase in upper alpha rhythm, particularly enhanced in elderly non-demented individuals, may imply that the genotype is related to preclinical dysregulation of hippocampal neurophysiology in aging and that this factor may contribute to the pathogenesis of AD.

## INTRODUCTION

Alzheimer’s disease (AD) is the major cause of dementia in the elderly. It is estimated that 35.6 million people worldwide currently suffer from dementia, with the prevalence projected to increase to 65.7 million by 2030 and 115.4 million by 2050. Two-thirds of these people will likely develop AD (). The incidence and prevalence of AD begins to rise as individuals reach the age of 65, so that by the time they are in their 80s and 90s the risk of clinical dementia is nearly 50%.

Alzheimer’s disease has a strong genetic basis with heritability estimates of up to 80% (Gatz et al., 2006). Mutations in the amyloid precursor protein gene (chr21), presenilin 1 (chr14), and presenilin 2 (chr1) genes are causative factors for familial AD (Goate et al., 1991; Levy-Lahad et al., 1995; Rogaev et al., 1995; Sherrington et al., 1995). A common polymorphism in the apolipoprotein E gene (*ApoE*), located on chromosome 19, has been established as the most common genetic risk factor for AD in Caucasian ethnic groups, including the Russian population (Saunders et al., 1993; Schmechel et al., 1993; Farrer et al., 1997; Rogaev, 1999).

Recent genome-wide association studies (GWAS) studies have provided evidence that polymorphisms of the clusterin (*CLU)* (chr8) and *PICALM* (chr11) genes are also associated with AD risk (Harold et al., 2009; Lambert et al., 2009; Golenkina et al., 2010). Carriers of the *CLU* rs1113600 *C* allele have 1.16 greater odds of developing late-onset AD than carriers of the potentially protective *T* allele. Although the AD-association with *CLU* polymorphism alone was not confirmed in some studied populations, the putative epistatic interaction of the *CLU* genotype with *APOE ε4* in risk for AD has been demonstrated (Golenkina et al., 2010). Approximately 36% of Caucasians carry two copies of the risk-conferring allele (Bertram et al., 2007), which imply significance of this gene for public health.

The *CLU* gene encodes glycoprotein clusterin, also known as apolipoprotein J, which shares several properties with ApoE. Clusterin and ApoE both act as amyloid-β (Aβ) chaperones to alter Aβ aggregation and/or clearance (Killick et al., 2012; Ling et al., 2012). Clusterin and ApoE are involved in the transport of cholesterol and phospholipids, and modulate AD-related pathways such as inflammation and apoptosis (Bettens et al., 2012; Ling et al., 2012). Clusterin is upregulated during different physiological and pathological states, such as senescence, type-2 diabetes mellitus, AD, and in various neoplasms (Song et al., 2012; Tang et al., 2013).

In order to identify early preclinical markers for AD, it is vital to find specific genotype–phenotype characteristics in individuals with hereditary risk for AD at different stages of the pathological process, including the preclinical period. Such biomarkers can be helpful for estimating the effect of potential therapies for preventing or delaying onset of neurodegenerative diseases (Illarioshkin et al., 2004; Feigin et al., 2007; Masdeu et al., 2012; Suslina, 2012). At present, there is still a mismatch between the known genetic factors of AD, and the biomarkers reflecting the development of the pathological process.

Electroencephalography (EEG) patterns are considered to be valuable as an endophenotype – a more basic biological trait that more directly reflects the influence of the genome (Gottesman and Gould, 2003). The heritability of EEG patterns has been shown to be in the range 70–90% (van Beijsterveldt et al., 1996). Multiple genes may modulate the alpha phenotype. Recent studies indicated that the catechol-*O*-methyl transferase (*COMP)* genotype and the gene encoding gamma-aminobutyric acid B (GABA_B_) receptor both influence alpha voltage (Enoch et al., 2003; Winterer et al., 2003; Bodenmann et al., 2009).

Testing the association of the AD risk alleles with EEG endophenotypes can help understand where in the brain, in which stage, and during what type of information processing the genetic variant has a role.

Quantitative EEG (qEEG) has been shown to be a reliable diagnostic tool in dementia research (Stam et al., 2003; Jeong, 2004; Babiloni et al., 2006b, 2011a, 2014; Dauwels et al., 2010; Moretti et al., 2012a). Slowing of EEG in AD is a uniform finding. Patients with mild AD are characterized by higher delta and theta, and lower alpha and beta power than normal elderly subjects (Huang et al., 2000; Lizio et al., 2011). In patients with mild cognitive impairment (MCI), which is considered to be a prodromic stage of AD, EEG parameters have presented magnitudes intermediate between those observed in normal subjects and in AD patients (Babiloni et al., 2006b). Longitudinal studies have revealed qEEG-based predictors of future decline in patients with MCI and even in normal elderly subjects (Prichep et al., 2006; Van der Hiele et al., 2008; Babiloni et al., 2011b).

Alterations of alpha rhythm in particular were found to be related to AD development. In a resting-state condition, posterior alpha rhythms showed a power decrement in patients with MCI as compared with healthy elderly subjects (Huang et al., 2000; Jelic et al., 2000; Koenig et al., 2005; Babiloni et al., 2006b, 2014). It has been reported that, in contrast to the decrease of alpha1 (6.9–8.9 Hz) and alpha2 (8.9–10.9 Hz) relative power, the alpha3 (10.9–12.9 Hz) relative power increased in patients with MCI (Moretti et al., 2007, 2011, 2012a,b).

Recent studies have demonstrated the association between the AD genetic risk variant *ApoE ε4* and EEG in patients with AD, MCI, and healthy subjects (Jelic et al., 1997; Lehtovirta et al., 2000; Babiloni et al., 2006b; Ponomareva et al., 2008, 2012; Lee et al., 2012). It was shown that AD patients carrying the *ApoE ε4* genotype have lower alpha power and lower alpha coherence as compared to non-carriers (Jelic et al., 1997; Lehtovirta et al., 2000; Ponomareva et al., 2008). Similarly, alpha1 and alpha2 sources in occipital, temporal and limbic areas as examined by LORETA was demonstrated to have lower amplitude in AD and MCI patients with *ApoE ε4* genotype compared with those non-carrying *ApoE ε4* (Babiloni et al., 2006b). The authors suggested that these neurophysiological abnormalities might reflect greater impairment of the cholinergic basal forebrain, hippocampal, and thalamocortical networks. In young healthy women, Lee et al. (2012) noticed a consistent trend across the brain, in which *ApoE ε4* carriers possessed lower regional power at the alpha band.

The effect of *CLU* polymorphism on EEG characteristics has not been previously investigated, although several morphofunctional alterations associated with the *CLU* gene risk variant were recently identified. Young healthy carriers of *CLU C* allele demonstrated lower white matter integrity in multiple brain regions, including several which are known to degenerate in AD (Braskie et al., 2011). Elderly cognitively normal carriers of the *CLU* risk allele showed significant dose-dependent longitudinal increases in resting-state regional cerebral blood flow (rCBF) in the brain regions intrinsic to memory processes, and faster rates of decline in verbal memory performance scores (Thambisetty et al., 2013). EEG activity and alpha rhythm in particular are closely related to the rCBF (Jann et al., 2010).

The purpose of this study was to examine the possible effects of the *CLU* genotype on resting-state alpha activity in non-demented adults and to estimate whether this effect is modified over the course of aging.

We tested the hypothesis that healthy adult carriers of the AD risk variant *CLU* *C* (homozygous *CLU* *CC* genotype) would show age-dependent alpha-rhythm alterations relative to carriers of the protective *T* allele (heterozygous *CLU* *CT* and homozygous *CLU* *TT* genotypes).

## MATERIALS AND METHODS

### PARTICIPANTS

The enrolled cohort included 87 non-demented individuals (33 men and 54 women, age range 20–80 years). All subjects were of Russian origin from Moscow and the Moscow region. Participants underwent a neurological examination and cognitive screening. The recruited subjects were free of dementia and other medical, psychiatric, and neurological conditions. Exclusion criteria included a personal history of mental illness, signs of clinical depression or anxiety, physical brain injury, neurological disorder, or other medical condition (e.g., hypertension, diabetes, cardiac disease, and thyroid disease), and a personal history of drug or alcohol addiction. The Spielberger state-trait anxiety inventory (Spielberger, 1983) and Hamilton rating scale for depression (Hamilton, 1960) were used to examine anxiety and depression. Subjects were evaluated with the mini-mental state examination (MMSE) and Clinical Dementia Rating (CDR) scale (Hughes et al., 1982). Only subjects with MMSE scores of 28 and more and CDR scale 0 cases were included in the study. All subjects were right-handed.

Informed written consent was obtained from all participants. The experimental protocol of this study was approved by the local Ethics Committee.

*ApoE* genotyping was performed on all participants, and to exclude the effect of the *ApoE* genotype on EEG characteristics, only subjects without the *ApoE ε4* allele were included in the study.

All subjects were divided into subgroups according to *CLU (CLU CC* and *CLUCT*&*TT*) polymorphism. The homozygous *CLU CC* group included subjects with two *C* alleles of *CLU*, and the *CLU CT*&*TT* group consisted of subjects with heterozygous *CLU CT* or homozygous *CLU TT* genotypes. The participants with *CLU CC* as well as with *CLU CT*&*TT* genotypes were also divided into cohorts of those younger and older than 50 years of age.

### EEG RECORDING

All recordings were obtained in the afternoon at 3–4 pm. During the experiments, the subjects sat comfortably in a chair. They were asked to close their eyes and to relax during the recording. The technician watched the subject’s vigilance state continuously by monitoring the EEG and observing the subject.

The registration and evaluation of EEG has been carried out in accordance with the International Pharmaco-EEG Society (IPEG) guidelines (Versavel et al., 1995; Jobert et al., 2012). EEGs were recorded during resting with eyes closed on a Nihon Kohden 4217 G EEG using a time constant of 0.3 s. The 16 Ag/AgCl electrodes were placed according to the international 10–20 system at O2, O1, P4, P3, C4, C3, F4, F3, Fp2, Fp1, T6, T5, T4, T3, F8, and F7 positions. Linked ears served as the reference. Electrode impedance did not exceed 10 kΩ. During the recording, 180 s of EEG in resting conditions were simultaneously sampled at 256 Hz and stored on a computer for further analysis off-line. The records were digitally filtered with a band-pass filter of 1.0–45.0 Hz prior to analysis. Periods of artifact were eliminated from subsequent analysis. Identification and removal of artifacts (ocular, cardiac, muscular, sweating and respiratory, electrode movements) were performed by two expert electroencephalographists (P.N.V., M.D.D.) in accordance with criteria thoroughly described elsewhere (Moretti et al., 2003; Tatum et al., 2011; Jobert et al., 2012).

### DATA ANALYSIS

Thirty-six to forty artifact-free 4-s epochs of resting EEG were processed by fast Fourier transform. Absolute power for the frequencies of interest: alpha1 (7.5–8.99), alpha2 (9.00–10.99), alpha3 (11–12.99), and for the regions of interest (ROI): occipital (O2, O1), frontal 1 (F4, F3), frontal 2 (Fp2, Fp1), temporal 1 (T6, T5), and temporal 2 (T4, T3) were calculated.

These alpha band frequencies were chosen by averaging those used in previous relevant EEG studies on aging, genetic influences, and dementia (Babiloni et al., 2006a,b; Bodenmann et al., 2009; Moretti et al., 2012a,b). This allowed better comparison of our results with the previous literature on aging and genetics, but it did not account for individual alpha frequencies peak (Klimesch, 1999).

Log transformations of the absolute power of the various bandwidths in each derivation were calculated in order to compensate for data skewness, as recommended by John et al. (1980).

### GENETIC ANALYSIS

Genomic DNA was isolated from peripheral venous blood by the standard phenol–chloroform extraction methodology, or by using a Qiagen kit for DNA isolation. Genotyping was performed by polymerase chain reaction (PCR) and followed by restriction fragment length polymorphism (RFLP) analysis. Amplification was performed according to the manufacturer’s instructions using both the Tercyc DNA amplifier (DNA technology, Russia) and the GeneAmp PCR System 9700 Thermal Cycler (Applied Biosystems).

To genotype the *APOE* gene locus, the following oligonucleotide primers were used: 5′_CGGCTGGGCGCG_GACATGGAGGA and 5′_TCGCGGGCCCCGGC_CTGGTACAC. The PCR protocol was as follows: preliminary denaturation at 95°C for 4 min; 5 cycles: 95°C for 45 s, 54°C for 25 s, and 72°C for 30 s; and 30 cycles: 95°C for 5 s, 58°C for 15 s, and 72°C for 5 s; the last stage was performed at 72°C for 3 min. PCR products were then cleaved by *Hha*I or *BstHH*I (SibEnzyme, Russia) and restriction products were analyzed in 7.5% polyacrylamide gel.

The *rs11136000* polymorphism in *CLU* gene was tested with the following oligonucleotide primers: 5′_CTTTGTAATGATGTACCATCTACCC and 5′_AGGCTGCAGACTCCCTGAAT. The PCR protocol was as follows: preliminary denaturation at 95°C for 1 min and 35 cycles: 94°C for 30 s, 57°C for 30 s, and 72°C for 1 min. The last stage was performed at 72°C. The 645 bp PCR products were then cleaved by *Acs*I restriction endonuclease (SibEnzyme, Russia) and restriction fragments were analyzed in 2% agarose gel.

### STATISTICS

Differences in demographic scores between the groups (*CLU CC* young, *CLU CT*&*TT* young, *CLU CC* old, *CLU CT*&*TT* old) were tested using analysis of variance (ANOVA) for continuous variables (age, education), and the Mann–Whitney *U* test for categorical variables (sex).

Electroencephalography parameters from each group were tested for the normal distribution by the Wilk–Shapiro test, and in no cases were the data skewed. The significance of the differences between the log-transformed EEG parameters was estimated using repeated measures of ANOVA in the general linear model (GLM) separately for alpha1, alpha2, and alpha3 bands, with Genotype (*CLU CC* vs *CLU CT*&*TT*) and Age cohort (old vs. young) as between-subjects factors, and ROI: occipital (O2, O1), frontal 1 (F4, F3), frontal 2 (Fp2, Fp1), temporal 1 (T6, T5), temporal 2 (T4, T3), and hemisphere (right, left) as a within-subject factor. *Post hoc* comparisons for between-subject effects and within-subject effects were analyzed using the Duncan test, and the level of significance was set to *P* < 0.05 for *post hoc* comparisons.

## RESULTS

**Table 1** shows the demographic information for the participants. There were no differences in age and sex between the *CLU CC* and *CLU CT*&*TT*subgroups in either the young or the old subgroups and in the whole sample (*P* > 0.05). There were no significant differences in sex between the young and the old subgroups with the same *CLU* genotype.

**Table 1:** Demographic characteristics of participants.

	Young cohort Age range: 20–50		Old cohort Age range: 51–80		All participants Age range: 20–80	
	*CLU CC*	*CLU CT&*TT	*CLU CC*	*CLU CT&*TT	*CLU CC*	*CLU CT&*TT
N	17	24	15	31	32	55
Age, years	28.4 ± 1.7	32.7 ± 2.0	64.1 ±2.4	62.6 ± 1.3	45.1 ± 3.5	49.6 ± 2.3
Sex (men/women)	9/8	9/15	5/10	10/21	14/18	19/36
Education, years	14.9 ± 0.2	14.7 ± 0.1	15.1 ± 0.1	14.9 ± 0.2	15.0 ± 0.1	14.8 ± 0.1

*Data are presented as means and standard errors.*

### INFLUENCE OF AGING ON TOPOGRAPHIC DISTRIBUTION AND FREQUENCY OF ALPHA ACTIVITY IN HEALTHY ADULTS

The ANOVA revealed a significant effect of ROI on alpha1, alpha2, and alpha3 absolute power (for alpha1, *F*[4,332] = 111.97, *P* = 0.0000; for alpha2, *F*([4,332] = 195.96, *P* = 0.0000; for alpha3, *F*[4,332] = 178.36, *P* = 0.0000). *Post hoc* comparisons showed that in the entire sample, which included young and old cohorts, absolute power was higher in occipital than in frontal and temporal regions in the alpha1, alpha2, and alpha3 bands (*P* < 0.0001). Moreover, the power of all alpha bands was higher in frontal as compared to temporal areas (*P* < 0.0001).

**FIGURE 1:**
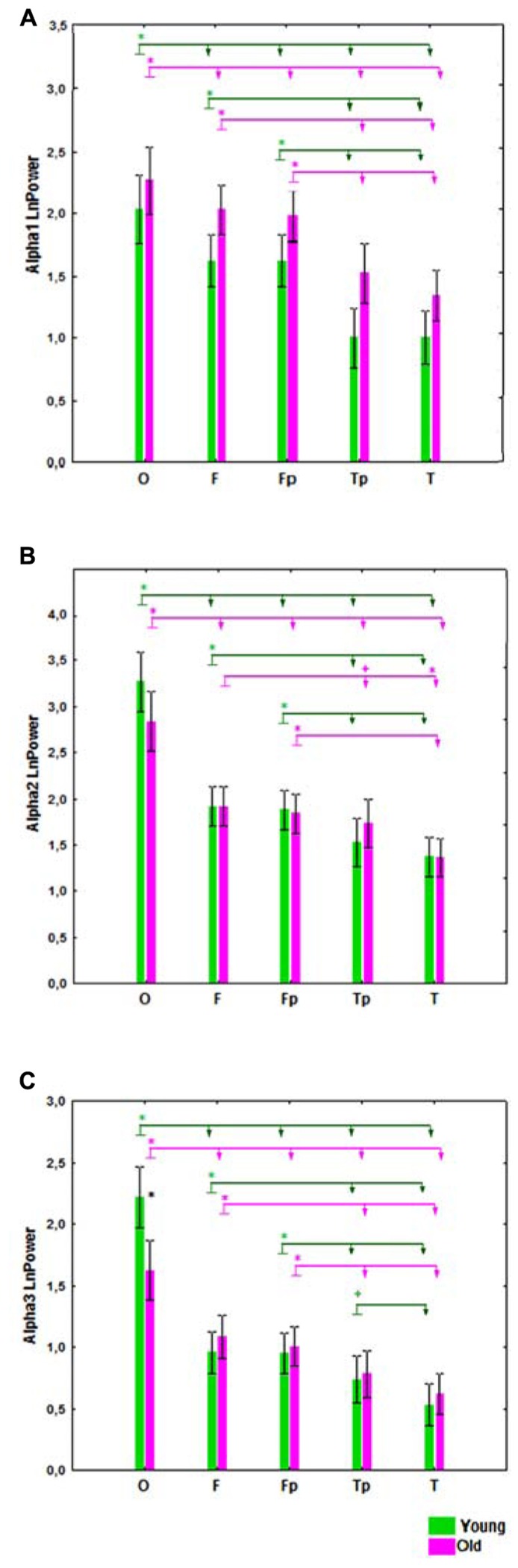
**Absolute power (mean and SE) of alpha1 (A), alpha2 **(B)**, and alpha3 **(C)** bands in the young and old cohorts, for occipital (O), frontal (F), frontal poles (Fp), temporal posterior (Tp), and temporal (T) areas.** Black asterisks (*) indicate a *P* < 0.01 significant difference in absolute spectral power between two cohorts in the same region of interest (ROI). The arrows labeled with green (for the young cohort) and purple (for the old cohort) asterisks compare different ROI in the same cohort. The ROI at the start of the arrow has either (+) *P* < 0.05 or (*) *P* < 0.01 significant differences in absolute spectral power compared the ROIs at the ends of the arrow.

There was no significant statistical Age × ROI interaction effect on alpha1 power (**Figure 1A**), but such an effect was observed on alpha2 and alpha3 bands (*F*[4,332] = 6.33, *P* = 0.00006 for alpha2; *F*[4,332] = 15.30, *P* = 0.00000 for alpha3). In the old cohort, the differences in alpha2 power between the ROI were reduced. Whereas in the young cohort, alpha2 power was higher in frontal Fp than in temporal posterior Tp areas (*post hoc* comparisons *P* = 0.002), in the old cohort the differences in these areas were not significant (*P* = 0.11). The differences between frontal Fp and temporal posterior Tp areas were significantly smaller in the old than in the young cohort (*P* = 0.02; **Figure 1B**).

Similarly, in the old cohort, the differences in alpha3 power between ROI were reduced as compared to the young cohort. In the young cohort, alpha3 power was higher in temporal posterior than in temporal areas (*P* = 0.02); in the old cohort these differences were not significant (*P* = 0.1), and age-related changes of these regional differences were also not significant. Alpha3 power was lower in the occipital ROI in the old cohort as compared to the young (*P* < 0.01; **Figure 1C**).

**FIGURE 2:**
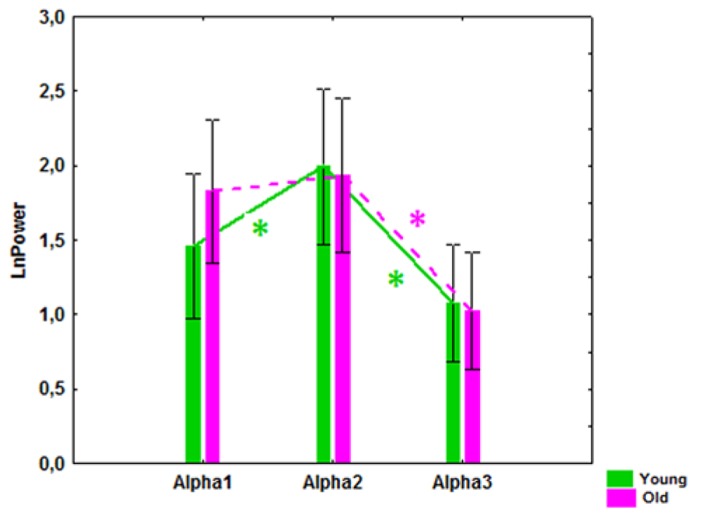
**Alpha1, alpha2, and alpha3 absolute power (mean and SE) in the healthy young and old cohorts.** *P í 0.01, significant differences between the alpha bands in the young (green) and old (purple) cohorts.

A significant interaction effect between the factors Age and Bands was observed (*F*[2,166] = 4.51, *P* = 0.01). In the young subjects, alpha2 power was significantly higher than alpha1 and alpha3 power (*P* = 0.00001), while in the old subjects the alpha1 power tended to increase and the differences between alpha1 and alpha2 power were not significant (**Figure 2**).

### STATISTICAL ANALYSIS OF CLU EFFECT ON ALPHA ACTIVITY

**FIGURE 3:**
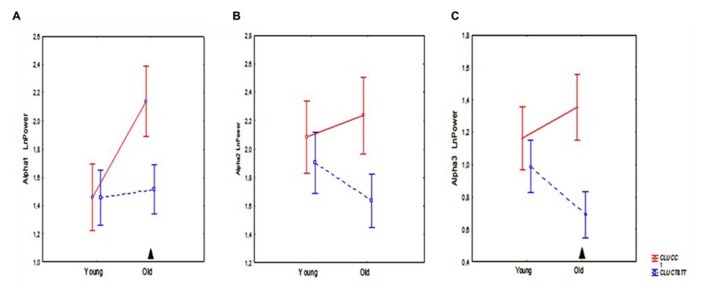
**The average absolute power of alpha1 (A), alpha2 **(B)**, and alpha3 **(C)** bands (mean and SE) in young and old subjects with *CLU CC* and *CLU CT*&*TT* genotypes.** Triangle indicates significant differences between the *CLU CC* and *CLU CT*&*TT* carriers *P* < 0.05.

The results of ANOVA showed that the main effect of *CLU* Genotype was significant on alpha3 (*F*[1,83] = 5.57, *P* = 0.021), but not on alpha1 (*F*[1,83] = 2.10, *P* = 0.15) or alpha2 (*F*[1,83] = 2.81, *P* = 0.10) absolute power. *Post hoc* comparison revealed that in the entire sample, which included old and young cohorts, alpha3 absolute power in the subjects with homozygous *CLU CC* genotype was significantly higher than in the subjects with heterozygous *CLU CT* and homozygous *CLU TT (CLU CT*&*TT)* genotypes (*P* = 0.017). Moreover, *post hoc* comparison showed that, in the old cohorts, alpha3 power was significantly higher in the CLU CC than in the *CLU CT*&*TT* carriers (*P* = 0.016), while in the young cohorts the differences in alpha3 power between the *CLU CC* and *CLU CT*&*TT* carriers did not reach a significant level (**Figure 3C**). There were no significant differences in alpha1 and alpha2 power between the young *CLU* *CC* and *CLU* *CT*&*TT* carriers (**Figures 3A,B**). In the old cohorts, alpha1 power was higher in the *CLU* *CC* than in the *CLU* *CT*&*TT* carriers (*P* = 0.04), while the differences of alpha2 power in the old *CLU* *CC* and *CLU* *CT*&*TT* carriers were not significant (*P* = 0.1; **Figures 3A,B**).

**FIGURE 4:**
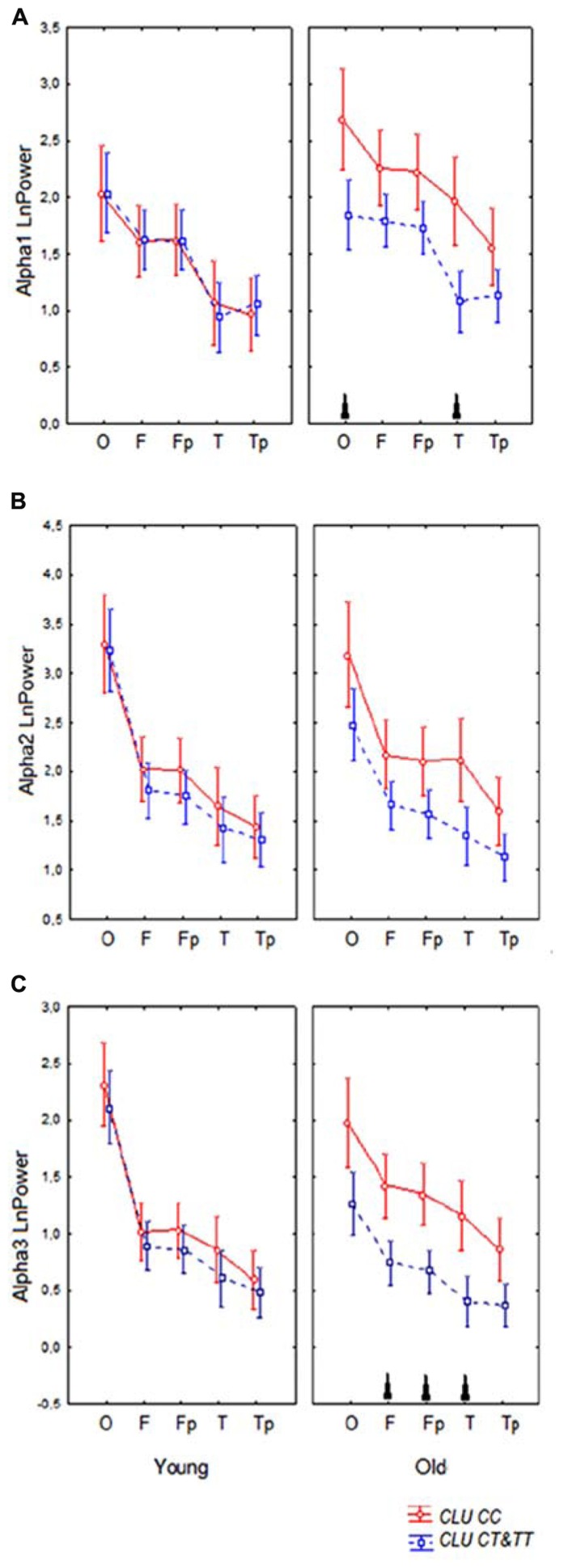
**Topographic distribution of alpha1 (A), alpha2 (B), and alpha3 (C) absolute power (mean and SE) in young and old carriers of *CLU CC* an *CLU CT*&*TT* genotypes in occipital O, frontal F, frontal poles Fp, temporal posterior Tp, and temporal T areas.** Triangle indicates significant differences between the *CLU CC* and *CLU CT*&*TT* carriers *P* < 0.05.

Topographic analysis demonstrated that the most pronounced differences between the homozygous *CLU CC* and *CLU CT*&*TT* carriers were observed in alpha3 power in the old cohorts. In the young cohorts, there were no significant differences in any ROI in alpha1, alpha2, and alpha3 power between *CLU CC* and *CLU CT*&*TT* carriers (**Figures 4A–C**). In the old cohort the differences between *CLU CC* and *CLU CT*&*TT* carriers were significant for alpha1 power in occipital (*P* = 0.02) and temporal posterior areas (*P* = 0.02), for alpha3 power – in frontal (*P* = 0.04), frontal poles (*P* = 0.03), and temporal posterior (*P* = 0.02) areas (**Figures 4A,C**).

A significant *CLU* × ROI interaction effect on the alpha1 power in the entire sample was observed (*F*[4,332] = 3.43, *P* = 0.009). In the *CLU CC* carriers alpha1 power was higher in occipital than in frontal areas (*P* < 0.0001) and in temporal posterior than in temporal areas (*P* = 0.001), while in the subjects with *CLU CT*&*TT* genotypes the differences in alpha1 power between occipital and frontal areas were smaller (*P* = 0.01), the differences between the temporal posterior and temporal areas were not significant (*P* = 0.3). There was a tendency toward higher alpha1 power in all ROI in the subjects with *CLU CC* genotype as compared to the subjects with *CLU CT*&*TT* genotype.

## DISCUSSION

The main findings of this study show that the *CLU* genotype exerts a significant effect on alpha absolute power in the resting-state EEG of healthy adults. The homozygous presence of the AD risk variant *CLU CC* in non-demented subjects was associated with an increase of alpha3 and to a lesser, though significant, extent of alpha1 power in the subjects older than 50 years of age. *CLU* genotype-related differences were also found in the topographic distribution of alpha1 activity: in the subjects with homozygous *CLU CC* genotype, alpha1 power was higher in occipital than in frontal regions, while in the subjects with heterozygous *CLU CT* and homozygous *CLU TT (CLU CT*&*TT*) genotypes the differences in alpha1 power between occipital and frontal regions were not significant. The present study also showed age-related alterations of the topographic distribution of alpha2 and alpha3 activities, and an age-related increase in power of alpha1 relative to alpha2, all of which occurred in the subjects with *CLU CC* as well as with *CLU CT*&*TT* genotypes.

Alpha rhythm reflects the activity of dominant oscillatory neural networks in resting adults and represents a basic functional feature of the working brain (Klimesch, 2012). Alpha oscillations have been associated with essential cognitive functions, such as memory, intelligence quotient, internal attention (Cooper et al., 2006; Klimesch, 2012), and inhibitory control of motor programs (Pfurtscheller et al., 2000; Başar, 2012).

Inhibitory processes underlie alpha synchronization (Klimesch, 2012). During the awake resting condition, the voltage of the alpha rhythms is inversely correlated with the cortical activation. Alpha rhythm is modulated by thalamocortical and corticocortical interactions playing role in the transmission of sensorimotor information between subcortical and cortical pathways, and the retrieval of semantic information from cortical regions (Steriade and Llinás, 1988; Brunia, 1999; Pfurtscheller and Lopes da Silva, 1999).

According to prior research in this area, alpha rhythm is not a unitary phenomenon. Upper alpha (11–13 Hz) is more involved in cortical processes related to the semantic memory and low alpha (8–11 Hz) is more involved in attentional demands (Klimesch, 1999). Different neural networks have been suggested as generating low alpha and high alpha frequency bands. The modulation of the low alpha was proposed to be related to the corticosubcortical mechanisms, such as corticothalamic, corticostriatal, and corticobasal, while the upper alpha band is affected to a greater extent by the hippocampus and other corticocortical interactions (Moretti et al., 2012a,b).

Recent study has shown an increase of the upper alpha power in patients with MCI and AD, when compared to normal elderly subjects (Moretti et al., 2012a,b). The increase in alpha3/alpha2 ratio in frontal and temporoparietal areas was associated with hippocampal atrophy in MCI (Moretti et al., 2007). The increase of alpha3/alpha2 ratio in subjects with MCI was suggested to reflect impairment of the anterior attentive mechanisms in subjects with MCI, in spite of the absence of overt clinical deficit (Moretti et al., 2012a,b). This increase was hypothesized to be due to a removal of excitatory, synaptic cholinergic inputs in intracortical networks, which would produce a decrease in synaptic efficacy and functional disconnection of cortical circuits (Steriade, 2006).

Healthy carriers of AD risk variant *CLU CC*, especially old subjects with this genotype, may have similar, though less pronounced, alterations underlying the increase of upper alpha activity to those found in MCI subjects. These alterations may include the dysregulation of excitatory synaptic inputs, especially cholinergic ones, in hippocampus and frontal intracortical networks. In the old *CLU CC* carriers we also found an increase in alpha1 power, though less pronounced, than in alpha3 power. These finding suggest that in the old *CLU CC* carriers the dysregulation may affect other mechanisms, such as corticothalamic, corticostriatal, and corticobasal ones, involved in low alpha generation (Moretti et al., 2012a,b).

Even normal aging is accompanied by a gradual loss of cholinergic function caused by dendritic, synaptic, and axonal degeneration as well as a decrease in trophic support. As a consequence, impairments in intracellular signaling and cytoskeletal transport may mediate cholinergic cell atrophy, finally leading to the known age-related functional decline in the brain, including aging-associated cognitive impairments (Schliebs and Arendt, 2011).

In line with previous studies, our results also demonstrated that in all individuals, independently of *CLU* genotype, aging is accompanied by changes in spectral power and topographic distribution of alpha activity (Tsuno et al., 2002; Babiloni et al., 2006a; Chiang et al., 2011). We found the decrease of alpha3 power in occipital areas, the reduction of the differences of alpha2 and alpha3 activity between posterior and anterior areas (anteriorization of alpha) and the trend toward the decrease of alpha2 power and increase of alpha1 power in the old cohort as compared to the young. It has been demonstrated that posterior cortical alpha rhythms decreases in magnitude during physiological aging (Babiloni et al., 2006a). A slowing of the alpha frequency peak in normal adults during physiological aging has also been reported (Klimesch, 1999). The anteriorization of alpha activity in elderly subjects was found to be related to a decreased level of vigilance (Tsuno et al., 2002). It was suggested that the anteriorization of alpha activity is related to the alterations in activation of posterior and anterior default mode networks (DMNs) and that these changes might be susceptible to dopaminergic influences (Knyazev, 2012). Chronic excessive neuronal activity during a resting-state condition in DMN can lead to Aβ deposition (Bero et al., 2011; de Haan et al., 2012). On the other hand, elevated level of Aβ elicits epileptiform activity, probably by enhancing synchrony among the glutamatergic synapses (Palop and Mucke, 2009). The brain regions of the DMN were shown to be preferentially vulnerable to neurodegenerative processes (Vlassenko et al., 2010; Hsiao et al., 2013).

The effect of aging on EEG is modulated by genetic factors (Babiloni et al., 2006b; Ponomareva et al., 2012). Several lines of evidence imply that the effect of *CLU* genotype on brain function may be observed before the onset of cognitive impairment. The *CLU* risk variant rs11136000 was found to be associated with reduced integrity of broad white matter regions, as observed with diffusion tensor imaging in young healthy adults (Braskie et al., 2011). fMRI study showed aberrant activation in the frontal and posterior cingulate cortex and the hippocampus during working memory performance in healthy young individuals carrying *CLU* AD risk genotype (Lancaster et al., 2011).

Recently the robust changes in rCBF in cognitively normal old individuals carrying the *C*-allele of the rs11136000 SNP were revealed (Thambisetty et al., 2013). These changes consisted of significant longitudinal increases in rCBF in the hippocampus and anterior cingulated cortex. The authors suggested that the effect of *CLU CC* genotype may be related to the deposition of beta amyloid, and that affected regions are vulnerable to disruption by deposition of beta amyloid, even in the non-demented elderly.

The effect of *CLU* genotype on resting EEG in healthy subjects was not similar to the effect of *ApoE* genotype found in prior studies (Babiloni et al., 2006b; Lee et al., 2012). The differences are in line with the differing influence of *CLU* and *ApoE* genotype on resting rCBF in normal aging (Thambisetty et al., 2013). The authors reported longitudinal increase during aging of resting-state rCBF in the hippocampus and anterior cingulate cortex in the *CLU CC* carriers and the decrease in resting rCBF in the frontal, parietal, and temporal cortices and its increases in the insular cortex in the old *ApoE ε4* carriers.

Alpha rhythm slowing was found to occur in aging, and the alpha1 band of the young group might have some functional differences from the alpha1 band in the old subjects (Chiang et al., 2011). This is a potential limitation of our study. However, as *CLU* genotype-related differences were found in the age-adjusted groups, this possible confounding factor could not affect the results concerning the influence of *CLU* genotype on alpha power.

## CONCLUSION

Our results show that the presence of the homozygous *CLU* *CC,* AD risk variant, is associated with increased absolute power of alpha3 activity and changes in topographical distribution of alpha1 activity and that this effect is more pronounced in the subjects older than 50 years of age. The increased synchronization of upper alpha activity may be related to the alterations in cholinergic hippocampal and cortical networks. The effect of *CLU* genotype on alpha activity can be superimposed to the other EEG alterations that occur across physiological aging.

## Conflict of Interest Statement

The authors declare that the research was conducted in the absence of any commercial or financial relationships that could be construed as a potential conflict of interest.
